# Conjugative Coupling Proteins and the Role of Their Domains in Conjugation, Secondary Structure and *in vivo* Subcellular Location

**DOI:** 10.3389/fmolb.2020.00185

**Published:** 2020-08-11

**Authors:** Itxaso Álvarez-Rodríguez, Begoña Ugarte-Uribe, Igor de la Arada, José Luis R. Arrondo, Carlos Garbisu, Itziar Alkorta

**Affiliations:** ^1^Department of Biochemistry and Molecular Biology, University of the Basque Country (UPV/EHU), Leioa, Spain; ^2^Instituto Biofisika (UPV/EHU, CSIC), University of the Basque Country (UPV/EHU), Spanish Research Council (CSIC), Leioa, Spain; ^3^NEIKER, Soil Microbial Ecology Group, Department of Conservation of Natural Resources, Derio, Spain

**Keywords:** coupling proteins, type IV secretion systems, bacterial conjugation, membrane proteins, antibiotic resistance spread

## Abstract

Type IV Coupling Proteins (T4CPs) are essential elements in many type IV secretion systems (T4SSs). The members of this family display sequence, length, and domain architecture heterogeneity, being the conserved Nucleotide-Binding Domain the motif that defines them. In addition, most T4CPs contain a Transmembrane Domain (TMD) in the amino end and an All-Alpha Domain facing the cytoplasm. Additionally, a few T4CPs present a variable domain at the carboxyl end. The structural paradigm of this family is TrwB_R388_, the T4CP of conjugative plasmid R388. This protein has been widely studied, in particular the role of the TMD on the different characteristics of TrwB_R388_. To gain knowledge about T4CPs and their TMD, in this work a chimeric protein containing the TMD of TraJ_pKM101_ and the cytosolic domain of TrwB_R388_ has been constructed. Additionally, one of the few T4CPs of mobilizable plasmids, MobB_CloDF13_ of mobilizable plasmid CloDF13, together with its TMD-less mutant MobBΔTMD have been studied. Mating studies showed that the chimeric protein is functional *in vivo* and that it exerted negative dominance against the native proteins TrwB_R388_ and TraJ_pKM101_. Also, it was observed that the TMD of MobB_CloDF13_ is essential for the mobilization of CloDF13 plasmid. Analysis of the secondary structure components showed that the presence of a heterologous TMD alters the structure of the cytosolic domain in the chimeric protein. On the contrary, the absence of the TMD in MobB_CloDF13_ does not affect the secondary structure of its cytosolic domain. Subcellular localization studies showed that T4CPs have a unipolar or bipolar location, which is enhanced by the presence of the remaining proteins of the conjugative system. Unlike what has been described for TrwB_R388_, the TMD is not an essential element for the polar location of MobB_CloDF13_. The main conclusion is that the characteristics described for the paradigmatic TrwB_R388_ T4CP should not be ascribed to the whole T4CP family. Specifically, it has been proven that the mobilizable plasmid-related MobB_CloDF13_ presents different characteristics regarding the role of its TMD. This work will contribute to better understand the T4CP family, a key element in bacterial conjugation, the main mechanism responsible for antibiotic resistance spread.

## Introduction

Type IV coupling proteins (T4CPs) are essential elements in the conjugative type IV secretion systems (T4SSs) and are also key elements in many pathogenic T4SSs. The members of this family display a high sequence, length, and domain architecture heterogeneity being the Nucleotide-Binding Domain (NBD) the only conserved domain in all T4CPs. For this reason, they are classified according to the different domain architectures: (i) VirD4-type subfamily that are integral membrane proteins; (ii) TraG-J pairs, which are also integral membrane proteins but additionally present a physical and functional association with another membrane protein of the T4SS; (iii) T4CPs without Transmembrane Domain (TMD), which could or could not interact with other T4SS membrane proteins creating a VirD4-type complex, like the pair TraJ_pIP501_ and TraI_pIP501_; (iv) FtsK-like T4CPs; and (v) Archaeal T4CPs (Llosa and Alkorta, 2017).

The structural paradigm of this family is the T4CP of conjugative plasmid R388, TrwB_R388_. It is a VirD4-type protein composed of a TMD at the N-terminus (consisting of two transmembrane α-helices connected through a small periplasmic loop) and a bulky globular cytosolic domain (CD). TrwB_R388_ is the only full-length T4CP that has been successfully purified to date (Hormaeche et al., 2002; Redzej et al., 2017), while trials for purifying other membrane T4CPs have not rendered the sufficient amounts of high quality protein for performing *in vitro* assays (Chen et al., 2008). For this reason most of the *in vitro* studies of T4CPs have been achieved using deletion mutant proteins that lack the TMD (Schroder and Lanka, 2003; Tato et al., 2007; Larrea et al., 2017). In this regard, the TMD deletion mutant protein of TrwB_R388_, TrwBΔN70, was resolved by X-ray crystallography, showing that the CD of TrwB_R388_ contains an NBD with the Walker A and Walker B motifs and a small membrane-distal All-Alpha Domain (AAD) (Gomis-Rüth et al., 2001).

Comparative studies of the properties of TrwB_R388_ and TrwBΔN70 showed significant differences regarding biological activity (such as *in vivo* function, *in vitro* nucleotide-binding properties, and *in vitro* ATPase activity), oligomerization pattern, subcellular location, and stability (Moncalián et al., 1999; Vecino et al., 2010, 2011; Hormaeche et al., 2002, 2004, 2006; Segura et al., 2013, 2014). For this reason it has been concluded that the TMD of TrwB_R388_ accomplishes a role beyond the anchorage of the protein to the membrane, influencing the location, stability, and activity of this protein.

To delve into the role of the TMD in T4CPs two different strategies have been followed. On the one hand, we have constructed a chimeric protein named TMD_TraJ_CD_TrwB_ composed of the TMD of TraJ_pKM101_, the phylogenetically closest T4CP to TrwB_R388_ from the conjugative plasmid pKM101 (Paterson et al., 1999; Alvarez-Martinez and Christie, 2009) and the CD of TrwB_R388_. This strategy has already been used for the study of components of T4SSs, showing interesting results (Bourg et al., 2009). Specifically, through a chimeric protein approach the function of the AAD of VirD4_At_ from the T-plasmid of *Agrobacterium tumefaciens* (Whitaker et al., 2016) and of the N-terminal HUH domain of TrwC_R388_ (Agúndez et al., 2018) have been analyzed. On the other hand, the T4CP from the mobilizable plasmid CloDF13, MobB_CloDF13_ and its deletion protein lacking the TMD, MobBΔTMD, have been constructed and studied. It is an interesting system to characterize since it is part of the rare MOB_C1_ plasmid family, which are mobilizable plasmids that encode their T4CP (Smillie et al., 2010). Additionally, MobB_CloDF13_ has been described as an atypical T4CP, due to its dual role in DNA transfer, since it acts as an accessory protein in CloDF13 relaxation process and also as a T4CP (Nuñez and de la Cruz, 2001).

We studied the functionality of these proteins in plasmid transfer, secondary structure, thermal stability, and subcellular localization. Our findings indicate that the TMD plays different roles in conjugative plasmid related and mobilizable plasmid related T4CPs. Specifically while the TMD could play a regulatory role in TrwB_R388_ this cannot be inferred from the results about MobB_CloDF13_.

## Materials and Methods

### Materials

n-dodecyl β-D maltoside (DDM) was purchased from Anatrace (Santa Clara, CA, United States). Mouse anti-His (C-term) monoclonal antibody and Alexa Fluor goat anti-mouse antibody were purchased from Invitrogen (Carlsbad, CA, United States) and Molecular Probes (Eugene, OR, United States), respectively. All buffers employed in this work are shown in Supplementary Table S1.

### Bacterial Strains and Bacterial Growth Conditions

*E. coli* DH5α strain was used as host for plasmid constructions. This strain also served as the donor for mating experiments by hosting the plasmids of interest. *E. coli* UB1637 served as the recipient for mating experiments. *E. coli* Lemo21 (DE3), *E. coli* BL21 (DE3), and *E. coli* BL21C41 (DE3) strains were used for protein production, purification, and *in vivo* localization.

*E. coli* strains were grown in LB medium and when necessary antibiotics were added at the following final concentrations: ampicillin (100 μg/mL), streptomycin (50 μg/mL), kanamycin (50 μg/mL), chloramphenicol (12.5–25 μg/mL), and thrimethoprim (10 μg/mL).

### Plasmids

The plasmids and oligonucleotides used in this study are listed in Tables 1, 2, respectively.

**TABLE 1:** Plasmids employed in this work.

Plasmid	Description	Phenotype	References
pET24a (+)	Expression vector	Kan^R^, C-terminal His tag	Novagen
R388	Natural plasmid	Tmp^R^, TRA_*W*_, IncW	Datta and Hedges, 1972
pKM101	Natural plasmid	Amp^R^, TRA_*N*_, IncN	Langer and Walker, 1981
pKM101Δ*traJ*	pKM101:Δ*traJ*	Amp^R^, TRA_*N*_, IncN, TraJ^–^	This work
pKM101Spc^r^_Δ*traJ*	pKM101:Δ*traJ* and Spt^R^ resistance cassette	Spt^R^, TRA_*N*_, IncN, TraJ^–^	Whitaker et al., 2016
pOPINE	Expression vector	Amp^R^, C-terminal His tag	Berrow et al., 2007
pOPINE-3C-eGFP	Expression vector	Amp^R^	OPPF-UK (Addgene)
pOPINE-3C-eGFP-*mobB*	pOPINE-3C-eGFP:*mobB*	pOPINE-3C-eGFP:MobB_CloDF13_GFP expression under T7 promoter	This work
pOPINE-3C-eGFP-*MobBΔTMD*	pOPINE-3C-eGFP:*MobBΔTMD*	pOPINE-3C-eGFP:MobBΔTMDGFP expression under T7 promoter	This work
pOPINE-*mobB*	pOPINE:*mobB*	pOPINE:MobB_CloDF13_ expression under T7 promoter	This work
pOPINE-*MobBΔTMD*	pOPINE:*MobBΔTMD*	pOPINE:MobBΔTMD expression under T7 promoter	This work
pSU1456	R388:Δ*trwB*	Su^R^, Tmp^R^, TRA_*W*_, IncW, TrwB^–^	Llosa et al., 1994
pSU4814	pSU19:*mob*_CloDF13_	Chl^r^, Rep (p15A), MOB (CloDF13)	Nuñez and de la Cruz, 2001
pSU4833	pSU4814:Δ*mobB*	Chl^R^, Rep (p15A), MobB^–^	Nuñez and de la Cruz, 2001
pUB9	pWaldo-GFPe:*trwB-GFP*	Kan^R^, TrwB-TEV-GFP-H_8_, expression under T7 promoter	Segura et al., 2014
pUBQ4	pET24a (+):*traJTMD-trwBCD*	Kan^R^, TMD_TraJ_CD_TrwB_-H_6_, expression under T7 promoter	This work
pUBQ4 (K142T)	pET24a (+):*traJTMD-trwBCD (K142T)*	Kan^R^, TMD_TraJ_CD_TrwB_-H_6_ containing the K142T mutation in the CD_TrwB_ domain, expression under T7 promoter	This work
pUBQ4GFP	pWaldo-GFPe:*traJTMD-trwBCD-GFP*	Kan^R^, TMD_TraJ_CD_TrwB_-TEV-GFP-H_8_, expression under T7 promoter	This work
pWaldo-GFPe	Expression vector	Kan^R^, C-Terminal GFP and His tag	Waldo et al., 1999

**TABLE 2:** Oligonucleotides used in this work.

Construct	Template	Protein	Sequence (5′ → 3′)		Cloning technology
pOPINE-*mobB*	pSU4814	MobB_CloDF13_	F:	AGGAGATATACCATGTTTAATACGGATTCGCTTGCCTGGCAGTGG	In-fusion^a^
			R:	GTGATGGTGATGTTTGTACAGCCCCGCAAAATCATCATCACCCG	
pOPINE-*MobBΔTMD*	pSU4814	MobBΔTMD	F:	AGGAGATATACCATGGACGAGGCGGTGAACGCGAAAC	In-fusion^a^
			R:	GTGATGGTGATGTTTGTACAGCCCCGCAAAATCATCATCAC	
pOPINE-3C-eGFP-*mobB*	pSU4814	MobB_CloDF13_GFP	F:	AGGAGATATACCATGTTTAATACGGATTCGCTTGCCTGGCAGTGG	In-fusion^a^
			R:	CAGAACTTCCAGTTTGTACAGCCCCGCAAAATCATCATCACCCG	
pOPINE-3C-eGFP-*MobBΔTMD*	pSU4814	MobBΔTMDGFP	F:	AGGAGATATACCATGGACGAGGCGGTGAACGCGAAAC	In-fusion^a^
			R:	CAGAACTTCCAGTTTGTACAGCCCCGCAAAATCATCATCAC	
pUBQ4 (K142T)	pUBQ4	TMD_TraJ_CD_TrwB_ (K142T)	F:	AAGCAACACCGATGTACCCGTACCAGTGGCA	Site-Directed mutagenesis^*b*^
			R:	GTGCCACTGGTACGGGTACATCGGTGTTGCTT	
pUBQ4GFP	pUBQ4	TMD_TraJ_CD_TrwB_GFP	F:	CCG**CTCGAG**ATGGACGATAGAGAAAGAGG	Restriction enzymes (*Xho*I and *Bam*HI)
			R:	CGC**GGATCC**GATAGTCCCCTCAAC	

*^a^Berrow et al. (2007). ^b^QuikChange II Site-Directed Mutagenesis (Stratagene, San Diego, CA, United States).*

pSU4814 and pSU4833 plasmids were kindly provided by Fernando de la Cruz. pOPINE (Addgene plasmid # 26043; RRID: Addgene_26043)^1^ and pOPINE-3C-eGFP (Addgene plasmid # 41125; RRID: Addgene_41125)^2^ plasmids were a gift from Ray Owens. pKM101Spc^r^_Δ*traJ* was kindly provided by Peter J. Christie. pKM101Amp^r^_Δ*traJ* plasmid was obtained by cleavage of the *spc cassette of* pKM101Spc^r^_Δ*traJ* plasmid using *Eco*RI restriction enzyme.

### Cloning of T4CPs

To construct the chimeric protein TMD_TraJ_CD_TrwB_ and the transmembrane deletion mutant protein of MobB_CloDF13_, MobBΔTMD, the sequences of TrwB_R388_, TraJ_pKM101_, and MobB_CloDF13_, were analyzed through bioinformatics tools. First, the different characteristics of the proteins, such as molecular weight and isoelectric point, were analyzed using *ProtParam*.^3^ Second, the topology of the membrane proteins was studied using *Topcons*^4^ (Tsirigos et al., 2015).

Then different constructions were achieved as follows:

TMD_TraJ_CD_TrwB_ chimeric protein consists of amino acids M_1_-D_76_ from TraJ_pKM101_ followed by amino acids L_71_-I_507_ from TrwB_R388_. The *tmd_TraJ_cd_TrwB_* sequence was synthesized *de novo* and inserted it into pET24a (+) plasmid vector using *Nde*I and *Xho*I restriction sites, rendering pUBQ4 plasmid to produce the chimeric protein. This construction was made by TOP Gene Technologies, Inc. (Saint-Lauren, QC-Canada). To study the role of the conserved lysine of the Walker A motif (K_142_), this residue was substituted with a threonine using the QuikChange II Site-Directed Mutagenesis Kit from Stratagene (San Diego, CA, United States) to obtain the TMD_TraJ_CD_TrwB_ (K142T) protein. Additionally, to clone the *tmd_TraJ_cd_TrwB_-eGFP* gene *tmd_TraJ_cd_TrwB_* sequence was inserted into the pWaldo-GFPe plasmid vector using *Xho*I and *Bam*HI restriction sites (Segura et al., 2014).

To clone MobB_CloDF13_-related proteins, the cloning of *mobB, MobBΔTMD, mob-eGFP*, and *MobBΔTMD-eGFP* genes was performed in the Oxford Protein Production Facility (OPPF-UK) using the High-throughput protocol described by Bird (2011). Specifically, MobBΔTMD soluble mutant protein consists of amino acids D_185_-Y_653_ of MobB_CloDF13_ obtained after deletion of amino acids M_1_-A_184_ from wild type MobB_CloDF13_.

All the oligonucleotides employed in the aforementioned cloning experiments are specified in Table 2.

### Overexpression and Purification

The same purification protocol was followed for TMD_TraJ_CD_TrwB_ and MobB_CloDF13_ proteins. Briefly, *E. coli* BL21C41 (DE3) cells freshly transformed with plasmids pUBQ4 for TMD_TraJ_CD_TrwB_ and pOPINE-*mobB* for MobB_CloDF13_ were grown overnight in LB (8 flasks of 10 mL) supplemented with the corresponding antibiotics at 37°C with continuous shaking. Then, cells were diluted 1:50 (v/v) with fresh LB supplemented with antibiotics (8 flasks of 500 mL) and were grown at 37°C with continuous shaking until an OD_600_ of 0.4–0.5 was achieved. Next, overexpression was induced by the addition of 1 mM isopropyl α-D-thiogalactopyranoside (IPTG) and cells were grown with continuous shaking at 25°C overnight. Cells were harvested by centrifugation at 8,000 *g* for 15 min at 4°C. The pellet was suspended in 80 mL of *Cell buffer*, frozen with liquid N_2_ and stored at −80°C.

For purification, cells were thawed at 37°C and 0.02 mg/mL DNase I, 1 mM dithiothreitol (DTT), 0.07% (w/v) lysozyme, 1 mM MgCl_2_, 1 mM phenylmethanesulfonyl fluoride (PMSF) and two tablets of cOmplete^TM^ EDTA-free Protease Inhibitor Cocktail from Sigma-Aldrich (San Luis, MO, United States) were added. From this point onward, the whole process was performed at 4°C to avoid aggregation of the proteins. After 45 min of incubation with agitation, cells were disrupted by sonication and centrifuged at 8,000 *g* for 15 min to remove non-lysed cell. The supernatant, containing the broken cells, was centrifuged at 138,000 *g* for 45 min to pellet the membrane fraction which was subsequently carefully resuspended in 30 mL of *Cell buffer*. Then DDM and NaCl were added to final concentrations of 19.6 mM and 600 mM, respectively, and the volume was adjusted to 40 mL. The mixture was incubated for 90 min with continuous stirring and then centrifuged at 138,000 *g* for 1 h.

The supernatant containing the protein to be purified was mixed 1:1 (v/v) with *MP1 buffer* to decrease the concentration of DDM and NaCl to 8.3 mM and 300 mM, respectively. Then, the sample was supplemented with 50 mM imidazole and loaded onto a 5 mL *HisTrap^TM^ FF* (GE Life Sciences; Marlborough, MA, United States) column previously equilibrated with *MP2 buffer*. To increase the binding of the protein, the sample was left recirculating overnight. Next, it was connected to an *ÄKTA-FPLC system* and it was washed with 50 mL of *MP2 buffer* at a flow rate of 2 mL/min until the absorbance at 280 nm reached the baseline. Bound proteins were eluted with *MP3 buffer*, at a flow rate of 1.5 mL/min and fractions of 1 mL were collected. Obtained samples were analyzed by SDS-PAGE and the ones containing each target protein were pulled-down and concentrated using a centrifugal filter with a MWCO of 100 kDa. Then, 5 mL of the resulting samples were separately loaded onto a *Superdex 200 HR 16/60* column and the size-exclusion chromatography (SEC) was performed in *MP purification buffer* at 0.5 mL/min. The fractions corresponding to each target protein were pulled-down and concentrated as explained before. Glycerol was added to a final concentration of 20% (v/v) and protein concentration was determined by measuring absorption at 280 nm. Finally, aliquots were stored at −80°C.

When TMD_TraJ_CD_TrwB_ was purified with the aim of performing infrared spectroscopy (IR) assays the DDM and NaCl concentrations of the MP purification buffer were changed to the ones described in the previously published purification protocols of TrwB_R388_ and TrwBΔN50 (i.e., 0.2 mM DDM and 200 mM NaCl instead of 0.6 mM DDM and 300 mM NaCl) (Vecino et al., 2011, 2012).

For MobBΔTMD purification, *E. coli* Lemo21 (DE3) cells freshly transformed with pOPINE-*MobBΔTMD* plasmid were grown in 4 L of LB supplemented with ampicillin at 37°C with continuous shaking until an OD_600_ of 0.5–0.6 was reached. Expression was induced with 1 mM IPTG and performed for 20 h at 25°C. Cells were harvested and stored as explained previously.

For protein purification, the cell suspension was thawed and the lysis protocol previously described for TMD_TraJ_CD_TrwB_ and MobB_CloDF13_ was followed. Then, the sample was centrifuged at 138,000 *g* for 45 min to pellet the membrane fraction and the inclusion bodies. The supernatant with the soluble proteins was supplemented with 50 mM imidazole and loaded onto a 5 mL *HisTrap^TM^ FF* (GE Life Sciences; Marlborough, MA, United States) column, previously equilibrated with *MobBΔTMD1 buffer*. Affinity chromatography was performed as previously described for MobB_CloDF13_, but using *MobBΔTMD1 buffer* for washing and *MobBΔTMD2 buffer* for elution. Fractions containing the target protein were pulled-down and concentrated using a *Centricon YM-30* to a final volume of 600 μL. The resulting sample was centrifuged to remove aggregates and loaded onto a *Superdex 200 HR 10/30* column. The SEC was performed in *Cell buffer* at 0.3 mL/min and 0.5 mL fractions were collected. Fractions that contained the protein of interest were pulled-down and the sample was centrifuged to discard the aggregates. Glycerol was added to a final concentration of 20% (v/v) and aliquots were made.

### Mating Assays

Mating assays were performed as described by Llosa et al. (2003) with small modifications. Briefly, donors (*E. coli* DH5α co-transformed with the appropriate plasmids) and recipient cells (*E. coli* UB1637) were grown in LB supplemented with the corresponding antibiotics overnight at 37°C. For each mating assay 100 μL of the donor and the recipient cells were mixed, centrifuged, resuspended in 50 μL LB and placed onto a GS Millipore filter (0.22 μm pore size) settled on a pre-warmed LB-agar plate. After 1 h incubation at 37°C bacteria were washed from the filters in 2 mL LB by shaking at 450 rpm for 20 min and vortexing for 30 s. Then, 100 μL of the appropriate dilutions were plated on selective media for donors and transconjugants. The plates were incubated overnight at 37°C and the colonies were counted, normalizing the conjugation frequency as the number of transconjugants per donor cell.

### Infrared Spectroscopy

To accomplish IR studies of different T4CPs and their variants (i.e., TMD_TraJ_CD_TrwB_, MobB_CloDF13_, and MobBΔTMD) purification of each protein was carried out as previously described. The H-D exchange protocol was performed at 4°C and adapted for each protein. Briefly, TMD_TraJ_CD_TrwB_ was diluted with the IR buffer, dialyzed against the same buffer using a *D-Tube^TM^ Dialyzer Midi* (MWCO 3.5 kDa) (Merck; Darmstadt, Germany), diluted again in IR buffer and concentrated using an Amicon Ultra-0.5 mL centrifugal filter (MWCO: 100 kDa). A similar process was followed for MobB_CloDF13_ except for the dialysis step. Regarding MobBΔTMD, sample was diluted, dialyzed and concentrated as described for TMD_TraJ_CD_TrwB_ but using in MobBΔTMD IR buffer. Final protein samples were always above 1 mg/mL protein concentration.

Infrared spectra were recorded in a Thermo Nicolet Nexus 5700 (Thermo Fisher Scientific; Waltham, MA, United States) spectrometer equipped with a liquid nitrogen-refrigerated mercury-cadmium-telluride detector using a Peltier-based temperature controller (TempComp^TM^, BioTools; Wauconda, IL, United States), and a 25 μm optical path. Typically 370 scans for each, background and sample, were collected at 2 cm^–1^ resolution and averaged after each minute. Spectra were collected with OMNIC software (Nicolet) and data processing was performed with OMNIC and SpectraCalc, following previously resolved methods (Arrondo et al., 1993; Arrondo and Goñi, 1999).

The information about the secondary structure and about the thermal denaturation of T4CPs and their derivatives was obtained by IR spectroscopy through analysis of the infrared amide I band that corresponds mainly to the C = O stretching vibrations of the peptide bonds and which is located between 1700 and 1600 cm^–1^ region of the IR spectrum. Amide I band is conformationally sensitive and can be used to monitor the protein secondary structure composition and changes induced by thermal denaturation. Secondary structure studies were made at 20°C and band decomposition of the amide I was performed as previously reported (Vecino et al., 2011, 2012). For thermal stability studies samples were heated from 20 to 80°C at a rate of 1°C/min.

### Subcellular Location of T4CPs

Subcellular location of different T4CPs and their variants (i.e., TrwB_R388_, TMD_TraJ_CD_TrwB_, MobB_CloDF13_, and MobBΔTMD) was achieved by confocal fluorescence microscopy. To do so, two different approaches were used: (i) eGFP-labeling (Cormack et al., 1996) and (ii) immunofluorescence. Since the eGFP moiety only emits fluorescence when properly folded (Drew et al., 2005), the eGFP based approach allowed visualizing only properly folded proteins.

Prior to localization assays, the *in vivo* activity of the T4CP-eGFP fusion-proteins was proved by mating assays as previously described (Supplementary Table S2). Afterward, T4CP-eGFP fusion-proteins were expressed in *E. coli* BL21C41 (DE3) strain, except for MobBΔTMD that was expressed in BL21 (DE3) strain. To do so cells were transformed with pUBQ4, by induction with 1 mM IPTG at OD_600_ 0.4–0.5 for the membrane proteins and OD_600_ 0.5–0.6 for MobBΔTMD. Protein expression was performed for 4 and 20 h at 25°C. Additionally, the subcellular location of TrwB_R388_-related proteins was determined in the presence of pSU1456 plasmid, which codes for all the R388 conjugative proteins except TrwB_R388_. Similarly, MobB_CloDF13_ was also expressed in the presence of plasmid pSU1456 and to mimic the *in vivo* transfer of CloDF13, its location was additionally studied in the presence of plasmids pSU1456 (R388 plasmid that lacks TrwB_R388_ protein) and pSU4833 (CloDF13 plasmid that contains its mobilization region except for MobB_CloDF13_ protein). Sample handling was performed as described by Segura et al. (2014). The images were acquired in a Leica TCS SP5 confocal fluorescence microscope, with a 60× oil immersion objective. Sample excitation was performed with 488 nm wavelength, while fluorescence emission was measured between 505 and 525 nm. The images were analyzed using Huygens and ImageJ softwares. To ease the counting process and better distinguish the different locations the images were treated with the preset ICE filter of ImageJ software; in this manner five different locations for the T4CP-eGFP fusion-proteins were described (Supplementary Figure S1).

For immunofluorescence assays protein expression was performed as with the eGFP fusion-proteins. Sample collection and handling was performed as described by Segura et al. (2014). Cells were immunostained with mouse anti-His (C-term) monoclonal antibody as primary antibody, and Alexa Fluor goat anti-mouse as secondary antibody. Image acquisition was performed in an Olympus FluoviewTM 500 confocal fluorescence microscope at the “Analytical and high-resolution microscopy in biomedicine” facility (SGIker, UPV/EHU).

## Results

Bacterial conjugation is one of the main processes responsible for the horizontal dissemination of antibiotic resistance genes among bacteria. One of the essential proteins in this process is the T4CP, which is ubiquitous in all conjugative systems. Despite its importance, the only widely studied T4CP is TrwB_R388_. Given its central role in bacterial conjugation, detailed knowledge of the T4CP family could contribute to the development of new strategies against the spread of antibiotic resistance among bacteria.

Previously published papers have highlighted the role of the TMD on different characteristics of TrwB_R388_, such as plasmid conjugation (Moncalián et al., 1999), subcellular localization (Segura et al., 2014), nucleotide-binding (Hormaeche et al., 2006), hexamerization (Hormaeche et al., 2002; Matilla et al., 2010), protein stability (Hormaeche et al., 2004), interaction with other proteins of the T4SS of R388 (Segura et al., 2013), and ATP hydrolase activity (Tato et al., 2005, 2007). From all these studies it was inferred that the TMD of TrwB_R388_ has a role beyond the mere anchorage in the membrane.

To gain more knowledge about different T4CPs, and in particular about the role of their TMD in T4CP features, in this work a TrwB_R388_ chimeric protein that combines its CD with the TMD of its phylogenetically closest T4CP, TraJ_pKM101_, has been studied. Also one of the few T4CPs of mobilizable plasmids, MobB_CloDF13_, and its TMD deletion mutant protein, MobBΔTMD, have been studied. Plasmid transfer, secondary structure, thermal stability, and subcellular location studies have been carried out to shed light on the functioning of this protein family and in the role of their TMD.

### Cloning of Soluble Mutant and Chimeric Proteins

**FIGURE 1:**
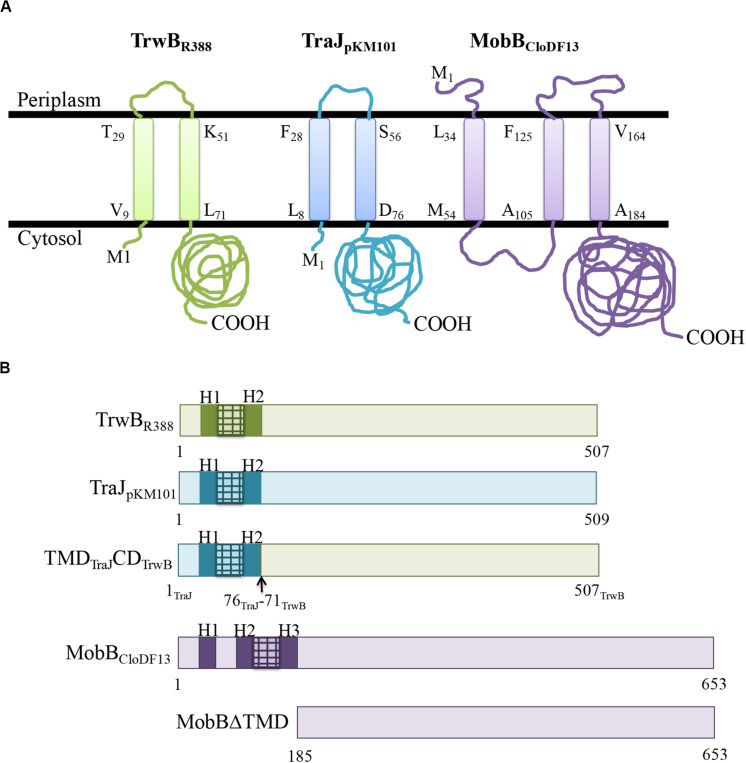
**(A)** Predicted membrane topology of TrwB_R388_, TraJ_pKM101_, and MobB_CloDF13_ proteins. Membrane topology of the different T4CPs was predicted using Topcons software. The black lines represent the inner bacterial membrane. M1, amino-terminus; COOH, carboxy-terminus. The first and last residues of each transmembrane helix are shown indicating their position in the sequence. Proteins from R388, pKM101, and CloDF13 plasmids are shown in green, blue, and purple, respectively. **(B)** Schematic representation of the different T4CPs and their variants used in the present study. Proteins from R388, pKM101, and CloDF13 plasmids are shown in green, blue and purple, respectively. The transmenbrane α-helices (H) and the small periplasmic loops connecting α-helices are indicated in dark boxes and stripped boxes, respectively.

The membrane protein topologies obtained after the bioinformatic analysis performed with Topcons software of TrwB_R388_, TraJ_pKM101_, and MobB_CloDF13_ are shown in Figure 1A. TrwB_R388_ and TraJ_pKM101_ have similar size and organization of their TMDs that consist of about 70 residues and contain two α-helixes connected by a small periplasmic loop. In contrast, the TMD of MobB_CloDF13_ is larger (about 150 amino acids) and is organized into three α-helixes. This information was used to design the chimeric and mutant proteins studied in this work (Figure 1B). The chimeric protein TMD_TraJ_CD_TrwB_ was made by combination of the TMD of the T4CP TraJ_pKM101_ and the CD of TrwB_R388_. In addition, in this work the T4CP of the mobilizable plasmid CloDF13, MobB_CloDF13_, and its TMD-deletion protein MobBΔTMD, were constructed (Figure 1B). The theoretical molecular weights of these proteins, necessary for their purification process, were calculated using *ProtParam*^3^ bioinformatic tool. The estimated molecular weights were 58.28, 73.95, and 53.13 kDa for TMD_TraJ_CD_TrwB_, MobB_CloDF13_, and MobBΔTMD, respectively. Finally, the eGFP fusion-proteins (i.e., TMD_TraJ_CD_TrwB_-eGFP, MobB_CloDF13_-eGFP, and MobBΔTMD-eGFP) were constructed and since they emitted a fluorescent signal, it was deduced that they were correctly folded (Drew et al., 2006).

### Functionality and Dominance Experiments

Through mating assays two different properties of TMD_TraJ_CD_TrwB_ were analyzed: (i) its capacity to complement the conjugative process in the absence of another T4CP (*functionality studies*) and (ii) its effect on each native conjugative system (R388 or pKM101 plasmids), being the corresponding T4CP present (TrwB_R388_ or TraJ_pKM101_, respectively) (*dominance studies*). Results obtained in mating assays are summarized in Table 3.

**TABLE 3:** Conjugation and dominance experiments with TMD_TraJ_CD_TrwB_. Transfer frequencies of plasmids pSU1456 and pKM101Δt*raJ* complemented with TMD_TraJ_CD_TrwB_ protein have been studied.

Plasmids in donors	T4CP	Transfer frequency
R388 (+)	Wild type TrwB_R388_	^*a*^2.1 10^–1^
R388Δ*trwB* (pSU1456) (−)	Ø	< 10^–8^
pKM101 (+)	Wild type TraJ_pKM101_	^b^2.2 10^–1^
pKM101Δ*traJ* (−)	Ø	< 10^–8^
R388Δ*trwB* (pSU1456) pUBQ4	TMD_TraJ_CD_TrwB_	1.82 10^–4^
pKM101Δ*traJ* pUBQ4	TMD_TraJ_CD_TrwB_	< 10^–8^
R388Δ*trwB* (pSU1456) pUBQ4 (K142T)	TMD_TraJ_CD_TrwB_ (K142T)	< 10^–8^
R388 pUBQ4	Wild type TrwB_R388_ TMD_TraJ_CD_TrwB_	1.13 10^–2^
pKM101 pUBQ4	Wild type TraJ_pKM101_ TMD_TraJ_CD_TrwB_	1.10 10^–2^

*Additionally, the effect of the chimeric protein in the conjugative process of R388 and pKM101 has been analyzed (dominance assays, shaded in gray). E. coli DH5α and UB1637 strains were used as donor and recipient cells, respectively. Transfer frequencies were normalized to the number of transconjugants per donor and are the mean value of at least five independent experiments. (+) Positive control; (–) negative control; Ø: no T4CP. ^a^Data from Segura et al. (2013). ^b^Data from Whitaker et al. (2016).*

Our results showed that TMD_TraJ_CD_TrwB_ efficiently complemented the Δ*trwB* mutation in R388 transfer but to a lower rate than native R388 (0.21 vs. 1.82 10^–4^ transconjugants per donor, respectively). On the contrary, TMD_TraJ_CD_TrwB_ was unable to complement the Δ*traJ* mutation in pKM101 transfer (Table 3). These results are in agreement with the necessary specific interactions between the CD of the T4CP and its cognate relaxase for transfer to happen as reported previously (Cabezón et al., 1997; Hamilton et al., 2000; Llosa et al., 2003).

It has been reported that mutation of the conserved lysine in the Walker A motif rendered a transfer deficient mutant protein TrwBK136T (Hormaeche et al., 2006). Similarly, an equivalent mutant of the soluble protein TrwBΔN70, TrwBΔN70 (K136T), lacked ATPase activity (Moncalián et al., 1999), underlying the essential role of this amino acid in the activity of TrwB_R388_. Here we studied the effect of the equivalent point mutation in the Walker A domain, TMD_TraJ_CD_TrwB_ (K142T), on the transfer capacity of chimeric protein. As expected, TMD_TraJ_CD_TrwB_ (K142T) was unable to complement the Δ*trwB* mutation in R388 plasmid transfer (Table 3). This is in agreement with the crucial role of the K residue as it has been reported with homologous mutants in other T4CPs (Moncalián et al., 1999; Kumar and Das, 2002; Gunton et al., 2005).

Next, to accomplish dominance assays, the transfer frequencies of plasmids R388 or pKM101 in the presence of the cognate T4CP and the chimeric protein were measured. It was observed that TMD_TraJ_CD_TrwB_ reduced the transfer frequency of R388 or pKM101 by an order of magnitude (Table 3).

### Mobilization Experiments

CloDF13 mobilization experiments were achieved to know whether the TMD of MobB_CloDF13_ was essential for the mobilization of the plasmid as happens with TrwB_R388_ or not as it has been described for TcpA_*pcW*3_, whose TMD-less mutant can perform conjugation although at a lower frequency (Parsons et al., 2007). First of all, the transfer frequency of the mobilizable region of CloDF13 (plasmid pSU4814) mediated by the T4SS of R388 was analyzed. Afterwards, the complementation experiments were performed in the presence of both pSU4833 (the mobilizable region of CloDF13 without functional MobB_CloDF13_) and pSU1456 plasmid (encoding for R388 conjugative system except for TrwB_R388_). Results obtained in mobilization assays are summarized in Table 4. It was observed that cloned MobB_CloDF13_ was functional when the T4SS of R388 was used, in agreement with what has been previously published for TrwB_R388_ (Hormaeche et al., 2006). Similarly, the deletion of the TMD, MobBΔTMD mutant, rendered a non-functional phenotype as it occurs with other T4CP mutants that lack the TMD, such as TrwBΔN70 and PcfCΔN103 (Moncalián et al., 1999; Chen et al., 2008).

**TABLE 4:** Mobilization experiments.

Plasmids in donors	T4CP	Transfer frequency
R388Δ*trwB* (pSU1456) pSU4814 (+)	Wild type MobB_CloDF13_	2.42 10^–2^
R388Δ*trwB* (pSU1456) MOB_CloDF13_Δ*mobB* (pSU4833) (−)	Ø	<10^–8^
R388Δ*trwB* (pSU1456) MOB_CloDF13_Δ*mobB* (pSU4833) pOPINE-*mobB*	MobB_CloDF13_	1.75 × 10^–2^
R388Δ*trwB* (pSU1456) pSU4833	Ø	<10^–8^

*Transfer frequency of pSU4833 plasmid complemented with MobB_CloDF13_ or MobBΔTMD mediated by the secretion channel of R388 conjugative system has been studied. E. coli DH5α and UB1637 strains were used as donor and recipient cells, respectively. Transfer frequencies were normalized to the number of transconjugants per donor and are the mean value of at least five independent experiments. (+) Positive control; (−) Negative control; Ø: no T4CP.*

### Secondary Structure of T4CPs and Their Variants

Analysis of the secondary structure components of TMD_TraJ_CD_TrwB_, MobB_CloDF13_, and MobBΔTMD were performed by IR spectroscopy through analysis of the IR amide I band.

**FIGURE 2:**
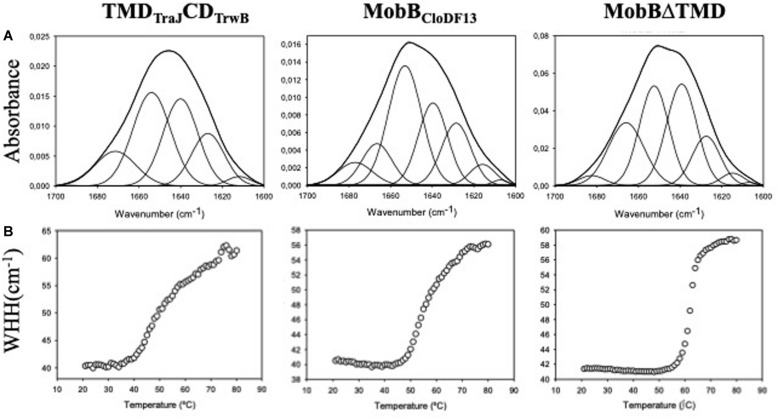
**(A)** Amide I region of the infrared spectra of TMD_TraJ_CD_TrwB_, MobB_CloDF13_, and MobBΔTMD. Proteins were purified, dialyzed against the corresponding buffer in D_2_O and analyzed by IR spectroscopy as explained in “Materials and Methods” section. Obtained spectra were curve-fitted to show the different secondary structure components as detailed in Table 5. **(B)** Thermal denaturation of TMD_TraJ_CD_TrwB_, MobB_CloDF13_, and MobBΔTMD as seen by IR spectroscopy. The widths at half-height (WHH) of the amide I bands are plotted as a function of temperature (°C). Thermal denaturation is marked by an abrupt increase in bandwidth. Mid-point denaturation temperature (Tm) values corresponding to each protein are detailed in Table 5.

The secondary structure of TMD_TraJ_CD_TrwB_ was compared to those of the native TrwB_R388_ and its mutants TrwBΔN50 and TrwBΔN70 (Vecino et al., 2012). Figure 2A shows the original spectra and the curve-fitting decomposition corresponding to TMD_TraJ_CD_TrwB_ purified in the presence of detergent. Band position, percentage area, and structure assignation corresponding to the deconvolved spectrum of the amide I region are summarized in Table 5 together with those previously reported of TrwB_R388_, TrwBΔN50, and TrwBΔN70 (Vecino et al., 2012).

**TABLE 5:** Secondary structure components and mid-point denaturation temperatures (Tm) of TrwB_R388_, TMD_TraJ_CD_TrwB_, TrwBΔN50, TrwBΔN70, MobB_CloDF13_, and MobBΔTMD.

	TrwB_R388_^a^		TMD_TraJ_CD_TrwB_		TrwBΔN50^a^		TrwBΔN70^a^		MobB_CloDF13_		MobBΔTMD	
Assignment	Position (cm^–1^)	Area (%)	Position (cm^–1^)	Area (%)	Position (cm^–1^)	Area (%)	Position (cm^–1^)	Area (%)	Position (cm^–1^)	Area (%)	Position (cm^–1^)	Area (%)
β-T + β-S	1676	5	1671	14	1676	2	1675	8	1677	7	1682	3
β-T	1661	27	/	/	1665	11	1665	11	1666	13	1665	22
α-H	1647	41	1654	35	1654	27	1653	26	1653	35	1652	29
Unordered + β-S	/	/	1640	30	1641	37	1640	39	1639	29	1639	30
β-S	1631	27	1626	17	1628	17	1627	12	/	/	/	/
Tm (°C)	48		51		48		63^b^		56		62	

*Secondary structure components were obtained from band decomposition of the amide I band of IR spectra in D_2_O medium. The spectra are shown in Figure 2. Only areas above 5% are shown. For details on assignments, see the text. ^a^Data from Vecino et al. (2011, 2012). ^b^Unpublished data from our group. β-T, β-turns; β-S, β-sheet; α-H, α-helix; Tm, mid-point denaturation temperature.*

The spectrum of TMD_TraJ_CD_TrwB_ purified in the presence of detergent exhibited four bands related to protein structure at 1671, 1654, 1640, and 1626 cm^–1^ (Table 5). Interpretation of the results must be done taking into account that band assignation is not always a straightforward process since its position can be altered by the environment (Arrondo and Goñi, 1999). The component at 1654 cm^–1^ was assigned to α-helix, the bands at 1626 and 1671 cm^–1^ were associated with the low and high-frequency vibrations of β-sheet, respectively, although it should be noted that the later is also assigned to β-turns. And the band at 1640 cm^–1^ was assigned to flexible, non-periodic elements.

When these results were compared to previously reported ones (i.e., TrwB_R388_ TrwBΔN50, and TrwBΔN70) (Vecino et al., 2012) it can be observed that the proportion of the α-helix (35%) is lower than the one observed in the native protein TrwB_R388_ purified in detergent (41%) and higher than the one shown in the deletion mutant proteins (26%). This result can be directly associated to the presence of a TMD both in TrwB_R388_ and TMD_TraJ_CD_TrwB_, even if in the later belongs to another T4CP such as TraJ_*pMK*101_. Regarding bands associated to β-sheet and β-turns, it is remarkable the absence of a band around 1661–1665 cm^–1^ in TMD_TraJ_CD_TrwB_, as it was observed in TrwB_R388_, TrwBΔN50, and TrwBΔN70. Nevertheless the total proportion of the different bands associated to β-sheet and β-turns of TMD_TraJ_CD_TrwB_ (31%) is similar to the proportion seen in the soluble mutant proteins and significantly lower than that of TrwB_R388_ (59%). Finally, a sizeable proportion (35%) of the structure of TMD_TraJ_CD_TrwB_ gave off a signal centered at 1640 cm^–1^ (assigned to flexible, non-periodic elements) as seen in the deletion mutant proteins TrwBΔN50 and TrwBΔN70 but not in TrwB_R388_ (Vecino et al., 2012). Previous studies about TrwBΔN70 and TrwBΔN50 showed that this band at 1640 cm^–1^ also had a β-sheet component (Vecino et al., 2012). And it was published that at higher temperatures the band at 1640 cm^–1^ split showing a β-sheet related band, which would not happen if the band was purely composed of unordered structures (Andraka et al., 2017). To elucidate if this also happened in TMD_TraJ_CD_TrwB_, IRS experiments at different temperatures were performed (20, 40, 60, and 80°C). With the increase of temperature, the 1640 cm^–1^ band shifts to 1645 cm^–1^ and that at 1626 cm^–1^ increases both its contribution and width, suffering also a shift to higher wavenumbers. This behavior indicates that although those two not resolved bands included in that at 1640 cm^–1^ do not directly split into two bands, there is a transfer of the β-sheet contribution to the 1626 cm^–1^ only β-sheet band, confirming that the 1640 cm^–1^ band in the chimeric protein was composed of both unordered and β-sheet elements.

With the aim of further studying if the deletion of the TMD has the same effect in all T4CPs, the same study was carried out with MobB_CloDF13_ and its deletion mutant MobBΔTMD to be compared with TrwB_R388_ and its variants. Figure 2A shows the original spectra and their curve-fitting decomposition corresponding to MobB_CloDF13_ purified in the presence of detergent and its deletion mutant MobBΔTMD. In this case, both proteins exhibited four main bands. Band position, percentage area, and structure assignation corresponding to the deconvolved spectrum of the amide I region are summarized in Table 5. It can be observed that the component at 1653 and 1652 cm^–1^ was assigned to α-helix in MobB_CloDF13_ and MobBΔTMD, respectively. But it should be pointed out that the proportion of the α-helix in MobBΔTMD (29%) was lower than in native protein (35%), as expected taking into account the three transmembrane α-helices postulated for the native protein (Figure 1A). The bands at 1666 or 1665 cm^–1^ were associated with β-turns which were also observed in the proteins studied in Vecino et al. (2012), but not in TMD_TraJ_CD_TrwB_. Nevertheless, proportions were slightly different (13 and 22% for MobB_CloDF13_ and MobBΔTMD, respectively). On the contrary, the signal centered at 1677 or 1682 cm^–1^ assigned to low-frequency vibrations of β-sheet and β-turns showed similar proportions in MobB_CloDF13_, MobBΔTMD (7 and 3%, respectively) and in the proteins studied in Vecino et al. (2012) but not in TMD_TraJ_CD_TrwB_ where the proportion of this component was 14%. Finally, as observed in TMD_TraJ_CD_TrwB_, TrwBΔN50 and TrwBΔN70, both MobB_CloDF13_ and MobBΔTMD had a band at 1639 cm^–1^ assigned to unordered structures that represented 30% of the structure, a band that is missing in TrwB_R388_.

### Thermal Stability of T4CPs and Their Variants

The information regarding the denaturation of TMD_TraJ_CD_TrwB_, MobB_CloDF13_, and MobBΔTMD was obtained through analysis of the amide I band (Figure 2B). Specifically, two bands appear at 1615–1620 cm^–1^ and 1680 cm^–1^ when the protein aggregates. The appearance of these bands allows monitoring the denaturation process of the protein and the calculation of the mid-point denaturation temperature (Tm) (Table 5). Data treatment and band decomposition of the original amide I have been described previously (Vecino et al., 2011, 2012).

The thermal stability of TMD_TraJ_CD_TrwB_ purified in detergent was compared to TrwB_R388_, TrwBΔN50, and TrwBΔN70 (Vecino et al., 2012). As observed in Figure 2B, the thermal denaturation of TMD_TraJ_CD_TrwB_ started at 35°C showing a mid-point denaturation temperature of 51.1°C at the tested conditions. This result is similar to TrwB_R388_ and to the TrwBΔN50 (Vecino et al., 2012; Table 5).

Thermal stability of MobB_CloDF13_ and MobBΔTMD was also studied by IR spectroscopy. As depicted in Figure 2B, the denaturation of the native protein starts at 47°C, achieving its Tm at 56°C, while the denaturation of MobBΔTMD starts at 55°C, achieving its Tm at 62°C (Table 5).

### Subcellular Location

Studies about subcellular location of different T4CPs reported up to now have rendered ambiguous results (Kumar and Das, 2002; Gunton et al., 2005; Segura et al., 2014). To gain a deeper knowledge of this matter, specifically regarding the role of the TMD, we have studied the subcellular location of TMD_TraJ_CD_TrwB_, MobB_CloDF13_, and MobBΔTMD under different experimental conditions. Subcellular location was analyzed by confocal fluorescence microscopy using eGFP-labeling and immunofluorescence techniques. Since eGFP proteins are only fluorescent when they are properly folded, their visualization ensures the analysis of functional proteins, excluding those that are denatured or included in inclusion bodies (Drew et al., 2006). In both studies similar results were obtained but eGFP-labeling rendered better quality images (Supplementary Figure S2).

**FIGURE 3:**
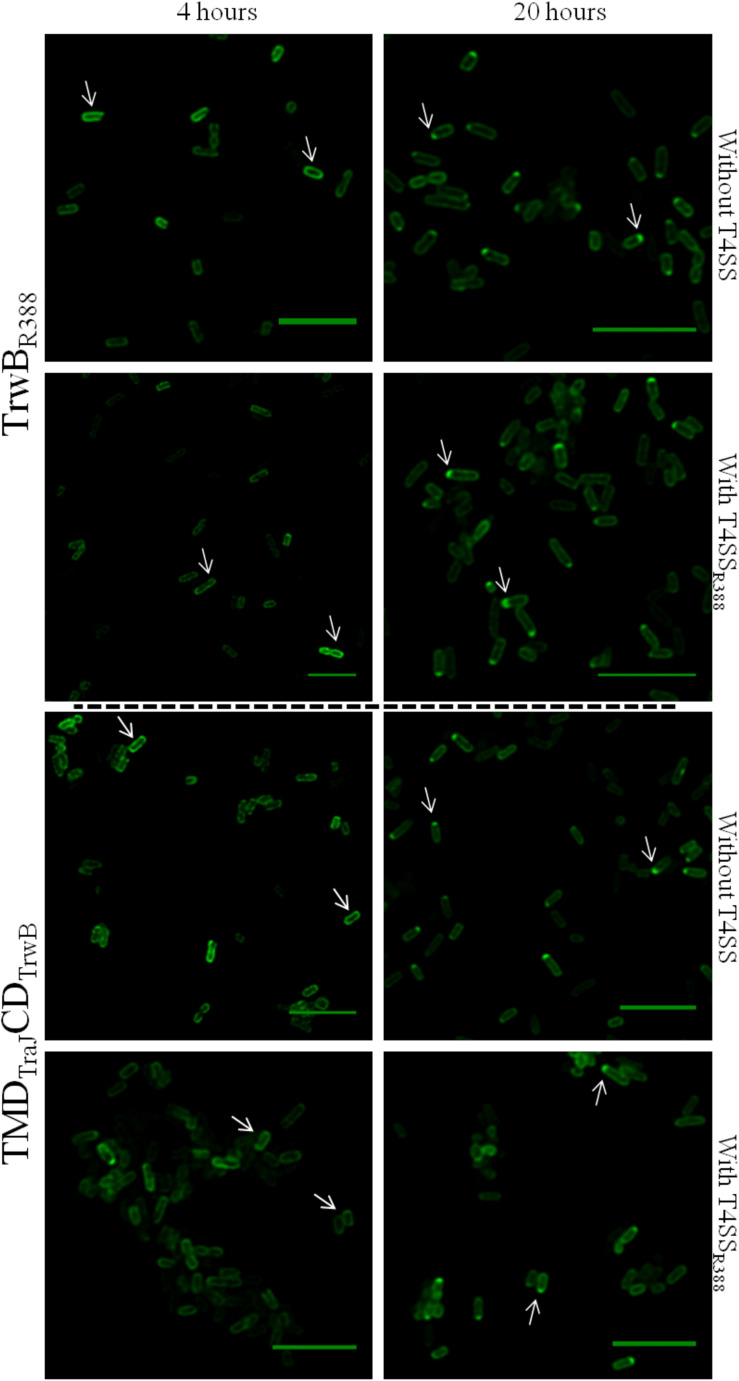
Subcellular location of TrwB_R388_GFP and TMD_TraJ_CD_TrwB_GFP fusion-proteins by confocal fluorescence microscopy. Proteins were expressed in *E. coli* BL21C41 (DE3) strain by induction with 1 mM IPTG for 4 (left panels) and 20 h (right panels) at 25°C. Subcellular location of TrwB_R388_GFP and TMD_TraJ_CD_TrwB_GFP was determined in *E. coli* strains containing plasmid pSU1456 that expresses all R388 conjugative proteins except TrwB_R388_, or without plasmid pSU1456. The images were acquired in a *Leica TCS SP5* confocal fluorescence microscope, with a 60× oil immersion objective. Sample excitation was performed with 488 nm wavelength, while fluorescence emission was measured between 505 and 525 nm. The images were analyzed using Huygens and ImageJ softwares. Arrrowheads indicate the eGFP fluorescence through the periphery (1st column) and at a single cell pole (2nd column). Scale bar: 5 μm.

**FIGURE 4:**
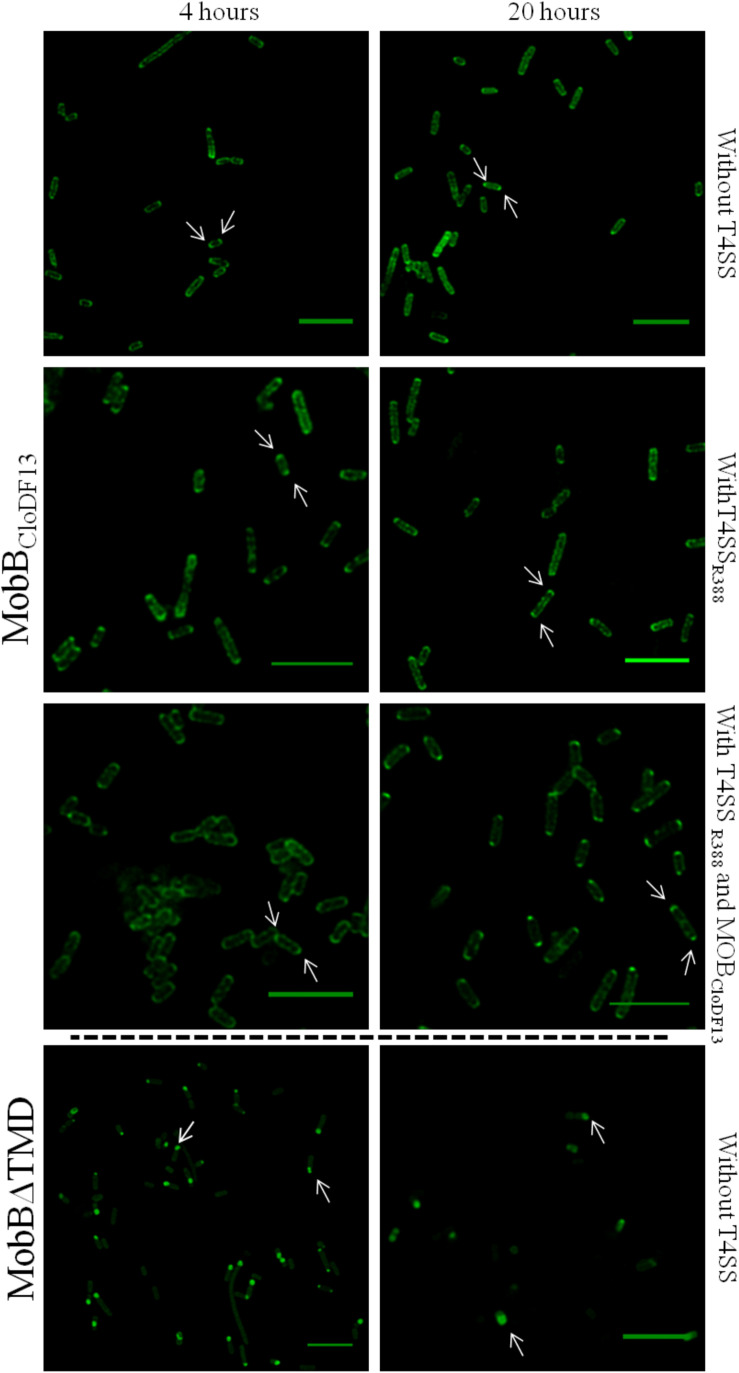
Subcellular location of MobB_CloDF13_GFP and MobBΔTMDGFP fusion-proteins by confocal fluorescence microscopy. MobB_CloDF13_GFP and MobBΔTMDGFP proteins were expressed in *E. coli* BL21C41 (DE3) and *E. coli* BL21 (DE3) strains, respectively. Subcellular location of these proteins was determined by induction with 1 mM IPTG after 4 (left panels) and 20 h (right panels) at 25°C. Additionally, subcelular location was analyzed in the presence of pSU1456 plasmid, which expresses all R388 conjugative proteins except TrwB_R388_, or without plasmid pSU1456. MobB_CloDF13_ was also expressed in the presence of both pSU1456 and pSU4833 that codes for the mobilization proteins of plasmid CloDF13 (MOB_CloDF13_), except for MobB_CloDF13_. The images were acquired in a *Leica TCS SP5* confocal fluorescence microscope, with a 60× oil immersion objective. Sample excitation was performed with 488 nm wavelength, while fluorescence emission was measured between 505 and 525 nm. The images were analyzed using *Huygens* and *ImageJ* software. Arrrowheads indicate the eGFP fluorescence foci at both cell poles (MobB_CloDF13_) and at a single cell pole (MobBΔTMD). Scale bar: 5 μm.

To study the effect of expression times in the absence of the rest of T4SS proteins, the subcellular location of each protein was visualized after induction with 1 mM IPTG for 4 or 20 h. Different patterns were observed in the location of each protein at different expression times (Figure 3 and Table 6). After 4 h of expression, TrwB_R388_ and TMD_TraJ_CD_TrwB_ were mainly located along the whole cell membrane and switched to a single-pole after 20 h, being this change less pronounced in the case of the chimeric protein. MobB_CloDF13_ was predominantly located at both poles both at 4 and 20 h, showing a little increase in one pole location after 20 h (Figure 4 and Table 6). On the contrary, most of the cells (95%) showed MobBΔTMD located on a single pole in the cytosol at both tested times.

**TABLE 6:** Subcellular location of different T4CP-eGFP fusion-proteins at different expression times in the absence or presence of T4SS_R388_.

	Expression time (h)	Without T4SS (%)			T4SS_R388_ (%)			T4SS_R388_ and MOB_CloDF13_ (%)		
		M	1P	2P	M	1P	2P	M	1P	2P
TrwB_R388_	4	**97 (77)**	11 (9)	18 (14)	**34 (84)**	3 (8)	3 (8)	–		
	20	31 (15)	**155 (75)**	29 (10)	10 (6)	**138 (90)**	6 (4)			
TMD_TraJ_CD_TrwB_	4	**85 (83)**	11 (11)	6 (6)	**55 (76)**	13 (18)	4 (6)	–		
	20	49 (40)	**62 (51)**	10 (8)	18 (14)	**85 (67)**	23 (18)			
MobB_CloDF13_	4	18 (18)	8 (8)	**73 (74)**	22 (19)	16 (14)	**80 (68)**	7 (5)	19 (13)	**122 (82)**
	20	20 (18)	24 (21)	**69 (61)**	16 (15)	16 (15)	**74 (70)**	7 (4)	43 (22)	**143 (74)**
MobBΔTMD^a^	4	0	**40 (95)**^b^	2 (5)^b^	N.d.			N.d.		
	20	0	**35 (95)**^b^	2 (5)^b^						

*The eGFP variants of the proteins in the first column were expressed in E. coli BL21C41 (DE3) strain by induction with 1 mM IPTG for 4 and 20 h. The numbers represent the amount of cells showing each location. Total number of cells counted for each sample was between 40 and 200. Numbers between parentheses represent the percentages calculated up to the total amount of cells counted for each sample. The main location for each condition is marked in bold. M, location through the perimeter of the cell membrane; 1P, location at a single-pole; 2P, location at both poles. –, without relevance for this study. N.d., not determined. ^a^Protein expressed in E. coli BL21 (DE3). ^b^Polar location in the cytosol.*

Since the interaction with other conjugative proteins of the T4SS could modify the subcellular location pattern (Segura et al., 2014), the proteins that were active *in vivo* (i.e., TrwB_R388_, TMD_TraJ_CD_TrwB_, and MobB_CloDF13_) were also observed in the presence of a T4SS lacking a functional T4CP that could interfere with the studied eGFP variant. On the one hand, TrwB_R388_ and TMD_TraJ_CD_TrwB_ were analyzed in the presence of the remaining conjugative proteins of R388 (i.e., in the presence of plasmid pSU1456 that lacks functional TrwB_R388_ but contains the remaining conjugative proteins). On the other hand, since CloDF13 needs the T4SS of a co-resident conjugative plasmid to be mobilized, the subcellular location of MobB_CloDF13_ was studied in the presence of R388 lacking functional TrwB_R388_ (plasmid pSU1456) and also in the presence of both, R388 lacking functional TrwB_R388_ (plasmid pSU1456) and the mobility region of CloDF13 lacking functional MobB_CloDF13_ (plasmid pSU4833) (Table 6). In these experiments (Figures 3, 4) the location pattern of each protein was the same in the absence or presence of a conjugative system (Table 6). However, although MobB_CloDF13_ kept two poles as its main location in all the tested conditions, in the presence of R388 lacking functional TrwB_R388_ it did not partially switch to a single-pole after 20 h, as in the absence of it.

The general observed pattern for all studied T4CPs was that the presence of T4SS enhanced the percentage of cells with the T4CP at the predominant location shown in the absence of the T4SS for each protein after 20 h (Figures 3, 4). Specifically, the percentage of cells showing TrwB_R388_ and TMD_TraJ_CD_TrwB_ at a single pole increased and so did the percentage of cells showing MobB_CloDF13_ at both poles. Moreover, regarding MobB_CloDF13_, the additional presence of its cognate mobilization region enhanced the effect produced by T4SS_R388_.

## Discussion

The increase of multidrug-resistant bacteria has become one of the major health concerns in our society (World Health Organization [WHO], 2019), being bacterial conjugation one of the key mechanisms responsible for the spread of antibiotic resistance genes among bacteria (Bello-López et al., 2019). This process is performed through a T4SS, a multiprotein complex that transfers the nucleoprotein substrate from a donor into a recipient bacterium (Waksman, 2019). T4CPs are essential proteins during conjugation, as they connect the substrates to be transferred in the cytosol with the secretion machinery in the membrane (Gomis-Ruth et al., 2005). Despite their importance, as membrane proteins are challenging to be studied, their characterization has been mostly accomplished using mutants that lack their TMD (Schroder and Lanka, 2003; Tato et al., 2007; Larrea et al., 2017). However, several studies performed with TrwB_R388_, the full-length T4CP of the conjugative plasmid R388, have proven that the TMD is more than a mere anchor to the membrane and that it has a role in protein activity, stability, oligomerization and subcellular localization (Moncalián et al., 1999; Hormaeche et al., 2002, 2004, 2006; Vecino et al., 2010, 2011; Segura et al., 2013, 2014).

The aim of this work has been to provide new data about different T4CPs that will contribute to infer general conclusions on their functioning to develop strategies to inhibit them and control the spread of antibiotic resistance genes. To do so, we studied the *in vivo* functionality, secondary structure, thermal stability, and subcellular location of TrwB_R388_, its chimeric protein TMD_TraJ_CD_TrwB_, and the T4CP of the mobilizable plasmid CloDF13, MobB_CloDF13_, and its TMD-less mutant, MobBΔTMD.

### *In vivo* Functionality

TMD_TraJ_CD_TrwB_ was able to complement the Δ*trwB* mutation for R388 transfer, although with a lower transfer frequency than TrwB_R388_. This decrease in conjugation frequency may be due to a combination of effects such as conformational changes in the CD of TrwB_R388_ due to its chimeric nature that render a less active protein and the heterologous interaction between TMD_TraJ_ and the T4SS of R388 (Llosa et al., 2003). Thus, TMD_TraJ_CD_TrwB_-mediated transfer of R388 would occur through a specific interaction between CD_TrwB_ with its cognate relaxase, TrwC_R388_, and an unspecific interaction of TMD_TraJ_ with the heterologous T4SS from R388. This could explain why TMD_TraJ_CD_TrwB_ did not complement the Δ*traJ* mutation (pKM101Δt*raJ*) since CD_TrwB_ could not recognize the heterologous TraI_pKM101_ relaxase, even if TMD_TraJ_ interacted with its cognate T4SS_pKM101_.

Moreover, TMD_TraJ_CD_TrwB_ showed negative dominance in the presence of the native T4CPs TrwB_R388_ or TraJ_pKM101_. Since it has been reported that TMD_TraJ_ can interact with both T4SS_R388_ and T4SS_pKM101_ (Llosa et al., 2003; De Paz et al., 2010; Celaya et al., 2017), a possible explanation for the observed negative dominance could be that competition for the T4SS occurred. According to this hypothesis, TMD_TraJ_CD_TrwB_ would have interacted with the secretion channel, sequestering it from interacting with the native T4CPs and reducing the conjugative rate of the wild type system. This hypothesis comes into agreement with the fact that the transfer of pKM101 using TraJ_pKM101_ and T4SS_R388_ is one order of magnitude lower than using T4SS_pKM101_ (Llosa et al., 2003). Another explanation compatible with dominance experiments with both systems would be that non-functional heteroligomers were made between the native and the chimera proteins, competing for the conjugative machinery and therefore lowering the transfer frequency. Any of these alternatives or a combination of them would have caused a decrease in the plasmid transfer rate, as observed in the dominance mating assays. It must be underlined that a point mutation in the Walker A domain, TMD_TraJ_CD_TrwB_ (K142T), resulted in a non-functional phenotype. Hence, the K mutation totally arrested transfer capacity as previously reported for other T4CPs (Moncalián et al., 1999; Kumar and Das, 2002; Gunton et al., 2005), suggesting that concerning its NBD TMD_TraJ_CD_TrwB_ is functionally similar to TrwB_R388_ despite the results obtained in the complementation and dominance studies.

Regarding CloDF13 system, it was observed that the deletion of the TMD rendered a non-functional MobBΔTMD as it occurs with other TMD-less mutants such as TrwBΔN70 and PcfCΔN103 (Chen et al., 2008). However, a TcpA mutant lacking the TMD, but not the N-term cytosolic residues, TcpA_Δ46–104_, was able to perform conjugation, although at a frequency lower than the wild type plasmid. In this regard, unpublished experiments with TrwBΔN8, which lacks the N-term cytosolic eight residues, showed a decrease in the transfer rate of more than three orders of magnitude (Vecino, 2009). Taken together these results, it seems that not only the TMD but also the N-term cytosolic residues have an important role in the transfer capacity of T4CPs. In this context, the importance of this small region in specific interactions with the relaxosome has already been described (Llosa et al., 2003; Schroder and Lanka, 2003). In the case of MobB_CloDF13_ its N-term is located in the periplasm (Figure 1A), which could be an important feature for recognition and interaction with conjugative secretion channels.

### Secondary Structure and Thermal Stability

As it has been described that the TMD influences the secondary structure of the CD and the thermal stability of TrwB_R388_ (Hormaeche et al., 2004; Vecino et al., 2011, 2012), in this work we have studied whether this behavior can be observed in TMD_TraJ_CD_TrwB_ and in MobB_CloDF13_.

Regarding TMD_TraJ_CD_TrwB_, one of the most important differences in comparison with TrwB_R388_ was the appearance of a band at 1640 cm^–1^, mainly assigned to flexible structures (non-periodic elements) related to a less compact overall structure (Echabe et al., 1998; Agopian et al., 2016). As the crystal structure of TrwBΔN70 shows flexible loops (Gomis-Rüth et al., 2002), the presence of the 1640 cm^–1^ band present at similar percentages (30–37%) in TMD_TraJ_CD_TrwB_, TrwBΔN50, and TrwBΔN70 mutant proteins but missing in TrwB_R388_, could be explained as the loss of the compact structure of TrwB_R388_ due to the deletion of its cognate TMD (Hormaeche et al., 2004).

Taking into account the values related to all the bands associated to β-sheet elements (1671, 1640, and 1626 cm^–1^) it could be concluded that the total percentage of β-sheets in TMD_TraJ_CD_TrwB_ is just slightly smaller to that of TrwB_R388_. Concerning β-turns, the band assigned to them in the TrwB_R388_ related proteins, 1661–1665 cm^–1^, was not observed in TMD_TraJ_CD_TrwB_. However, it can be postulated that the increase seen in TMD_TraJ_CD_TrwB_ of the band at 1671 cm^–1^, was partially related to the β-turns component seen in the mutants at 1665 cm^–1^. These would imply that the β-turns component of the chimeric protein is lower than the one of the native protein and similar to the TMD deletion mutants. Finally, TMD_TraJ_CD_TrwB_ shows a decrease in α-helix percentage (35%) in comparison with TrwB_R388_ (41%), but an increase in comparison to the mutants (26%).

Therefore it seems that TMD_TraJ_CD_TrwB_ presents qualitative and quantitative features in between the native protein and the deletion mutants. These results suggest that the presence of a heterologous full-length TMD does provide a more compact and ordered structure to the T4CP in comparison to the TMD-less mutants, even if it does not reach the level of the native protein. This result comes in agreement with the transfer capacity reduction of the chimeric protein that could be explained partially by the observed structural changes reported here.

To test if the results described for TrwB_R388_ could be extrapolated to other T4CPs, the secondary structures of MobB_CloDF13_ and MobBΔTMD were studied (Table 5). Surprisingly, MobBΔTMD presented an IR spectrum quite similar to the one obtained for MobB_CloDF13_. It presented smaller helical structure percentages and higher β-turns percentages but both showed similar unordered and β-sheet percentages. This suggests that in MobB_CloDF13_ the presence of the TMD does not have an effect on the structure of the CD as it does in TrwB_R388_. As MobB_CloDF13_ has to interact with heterologous T4SSs, it could be that its TMD has to interact with heterologous T4SSs without altering the structure of its CD where the specific interaction with its cognate relaxosome occurs.

Concerning the thermal denaturation of TMD_TraJ_CD_TrwB_, its Tm was similar to the ones described for TrwB_R388_ and the mutant lacking the first transmembrane helix, TrwBΔN50 (Table 5). This result suggests that although the secondary structures of the mutants differ from that of the native protein, their overall thermal stability is similar. Additionally, when comparing the results between both studied systems, as expected due to the high instability of purified membrane proteins (González Flecha, 2017) the soluble proteins showed higher mid-point denaturation temperatures than the full-length proteins. Specifically, MobBΔTMD and TrwBΔN70 showed similar Tm values (Tm 62 and 63°C, respectively); on the contrary, MobB_CloDF13_ was more stable than TrwB_R388_ against thermal denaturation (Tm 56 and 48°C, respectively). This could be related to different buffer compositions that had to be used when MobB_CloDF13_ and TrwB_R388_ were analyzed.

### Subcellular Location

The polar location of proteins in bacteria underlines their sophisticated internal organization, being important in many processes like chemotaxis and cellular division (Howard, 2004). Similarly, subcellular location has been considered important in bacterial conjugation (Chen et al., 2008; Leonetti et al., 2015). Sequence analysis, cell fractionation, and protein purification experiments proved that TrwB_R388_, TMD_TraJ_CD_TrwB_, and MobB_CloDF13_ are located in the bacterial membrane, while MobBΔTMD is located in the cytosol (data not shown). As studies in the literature do not show a consensus pattern either in the location of the T4CPS nor in the role of each domain in this property (Kumar and Das, 2002; Bauer et al., 2011; Segura et al., 2014), the subcellular location of TrwB_R388_, TMD_TraJ_CD_TrwB_, MobB_CloDF13_, and MobBΔTMD was investigated.

In this work we observed TrwB_R388_ located along the membrane after 4 h of induction and only after 20 h it focused at single pole. These results differ from our previous results where the polar location of TrwB_R388_ was observed after 4 h (Segura et al., 2014). However, the induction OD_600_ values were different (0.4 vs. 0.7, this work and previous work, respectively), probably rendering populations at different growth phase. On the contrary MobB_CloDF13_ was located at both poles in the membrane at 4 and 20 h after induction.

Previous studies have reported that in the absence of other conjugative proteins, T4CPs that lacked the whole TMD or even the periplasmic loop located in the cytosol or at the membrane periphery, respectively (Kumar and Das, 2002; Segura et al., 2014). Moreover, the TMD alone of TrwB_R388_ located at the membrane poles without the need for the CD (Segura et al., 2014), suggesting a leading role for the TMD in the subcellular location of the T4CPs. Surprisingly, MobBΔTMD located at a single cell pole in the cytosol even in the absence of other conjugative proteins. These results suggest that mobilizable plasmid-related T4CPs could use different mechanisms than VirD4-type T4CPs for subcellular location. It has been speculated that the polar location of T4CPs could be related to interactions with the cardiolipin enriched membrane poles (Mileykovskaya and Dowhan, 2009; Segura et al., 2014), but at the same time mediated by complex and dynamic changes in transduction, cytoskeleton proteins, etc. (Shapiro et al., 2002). Since interactions between mobilizable plasmid-related T4CPs and T4SSs are not specific, these T4CPs could have evolved to develop different mechanisms to interact with the membrane and ensure their polar location.

Previous studies (Kumar and Das, 2002; Gunton et al., 2005; Segura et al., 2014) have reported that the location of native T4CPs is independent of the presence of the rest of the conjugative proteins. Our results suggest that the presence of a complete conjugative system (i.e., mobilizable region and secretion channel) seems to enhance the polar location of wild type T4CPs. Moreover, although the location of MobB_CloDF13_ at both poles was enhanced in the presence of T4SS_R388_, it was further enhanced when MOB_CloDF13_ was also present.

Taking all together, it seems that that no universal location patterns can be attributed to T4CPs. Nevertheless, three conclusions can be undertaken regarding subcellular location: (i) T4CPs localize either at a single pole or both poles, depending on the system; (ii) the presence of a TMD is not essential for the polar location of a mobilizable plasmid associated T4CP, and (iii) the presence of a conjugative system enhances the polar location of full-length T4CPs.

To sum up, the comparative study between the conjugative system related TrwB_R388_ and the mobilizable plasmid-related MobB_CloDF13_ and their variants has underlined that the characteristics described for the paradigmatic conjugative plasmid related VirD4-type T4CPs and their TMDs should not be ascribed to the whole T4CP family.

## Data Availability Statement

The raw data supporting the conclusions of this article will be made available by the authors, without undue reservation, to any qualified researcher.

## Author Contributions

IÁ-R, CG, and IAl contributed to the design of the work (text and figures) and the acquisition of the data, writing and revision of the content, approval of the last version, and ensuring accuracy and integrity of the work. IAr and JA contributed to acquisition of the data, revision of the content, and approval of the last version of the work. BU-U contributed to writing, revision of the content, and approval of the last version. All authors contributed to the article and approved the submitted version.

## Conflict of Interest

The authors declare that the research was conducted in the absence of any commercial or financial relationships that could be construed as a potential conflict of interest.

## Footnotes

1 http://n2t.net/addgene:26043

2 http://n2t.net/addgene:41125

3 https://web.expasy.org/protparam/

4 http://topcons.cbr.su.se/
