# Supplementary material for: Conjugative Coupling Proteins and the Role of Their Domains in Conjugation, Secondary Structure and in vivo Subcellular Location
Source: Front Mol Biosci. 2020 Aug 11;7:185. doi: 10.3389/fmolb.2020.00185 (PMC7431656; doi:10.3389/fmolb.2020.00185)
Supplement: Supplementary file 1 [file Table_1.pdf]

Supplementary Material

Supplementary Figures and Tables

TABLE S1. Buffers employed in this work.

Buffer name	Composition
Cell buffer	50 mM Tris-HCl (pH 7.8), 0.1 mM EDTA, 200 mM NaCl
MP1 buffer	50 mM Tris-HCl (pH 7.8), 0.1 mM EDTA
MP2 buffer	50 mM Tris-HCl (pH 7.8), DDM 0.6 mM, 0.1 mM EDTA, 50 mM imidazole, 300 mM NaCl
MP3 buffer	50 mM Tris-HCl (pH 7.8), DDM 0.6 mM, 0.1 mM EDTA, 225 mM imidazole, 300 mM NaCl
MP purification buffer	50 mM Tris-HCl (pH 7.8), DDM 0.6 mM, 0.1 mM EDTA, 10% (v/v) glycerol, 300 mM NaCl
MobBΔTMD 1 buffer	50 mM Tris-HCl (pH 7.8), 0.1 mM EDTA, 50 mM imidazole, NaCl 200 mM
MobBΔTMD 2 buffer	50 mM Tris-HCl (pH 7.8), 0.1 mM EDTA, 225 mM imidazole, NaCl 200 mM
TMD$_{TraJ}$CD$_{TrwB}$ IR buffer	50 mM Tris-DCl (pD 7.8), DDM 0.2 mM, 0.1 mM EDTA, 200 mM NaCl
MobB$_{CloDF13}$ IR buffer	50 mM Tris-DCl (pD 7.8), DDM 0.6 mM, 0.1 mM EDTA, 300 mM NaCl
MobB$_{CloDF13}$ΔTMD IR buffer	50 mM Tris-DCl (pD 7.8), 0.1 mM EDTA, 200 mM NaCl

TABLE S2. *In vivo* activity of T4CP-eGFP fusion-proteins. Transfer frequencies of plasmid pSU1456 complemented with TMD$_{TraJ}$CD$_{TrwB}$-eGFP and plasmid pSU4833 complemented with MobB$_{CloDF13}$-eGFP or MobBΔTMD-eGFP were studied. *E. coli* DH5α and UB1637 strains were used as donor and recipient cells, respectively. Transfer frequencies were normalised to the number of transconjugants per donor and are the mean value of at least five independent experiments.

Plasmids in donor	T4CP	Transfer frequency
pSU1456<br>pUBQ4-eGFP	TMD$_{TraJ}$CD$_{TrwB}$-eGFP	3.33 x 10^{-6}
pSU1456<br>pSU4833<br>pOPINE-3C-eGFP-*mobB*	MobB$_{CloDF13}$-eGFP	1.53 x 10^{-3}
pSU1456<br>pSU4833<br>pOPINE-3C-eGFP-*mobBΔTMD*	MobBΔTMD-eGFP	$<10^{-8}$

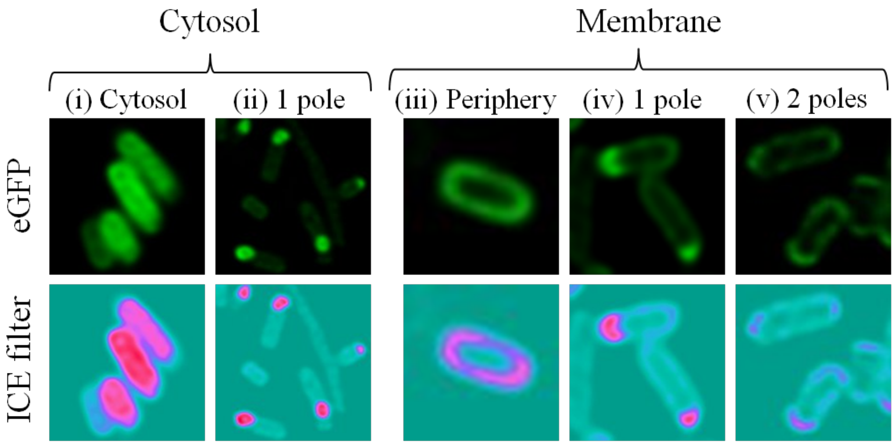

FIGURE S1. Different locations of T4CP-eGFP fusion proteins. Depending on the subcellular location five different protein locations were described: (i) in the cytosol; (ii) at the single cytosolic pole; (iii) throughout the membrane; (iv) at a single pole in the membrane; (v) at both poles in the membrane. The upper lane shows the eGFP and the lower lane shows the ICE filter of Image J software that was used to count. Images were taken of the following samples: (i) 4 hours after induction of eGFP (positive control); (ii) 4 hours after induction of MobBΔTMDGFP; (iii) 4 hours after induction of $TrwB_{R388}$GFP; (iv) 20 hours after induction in the presence of the plasmid $TrwB_{R388}$GFP pSU1456; (v) 4 hours after induction in the presence of the plasmids $MobB_{CloDF13}$-eGFP pSU1456 and pSU4833

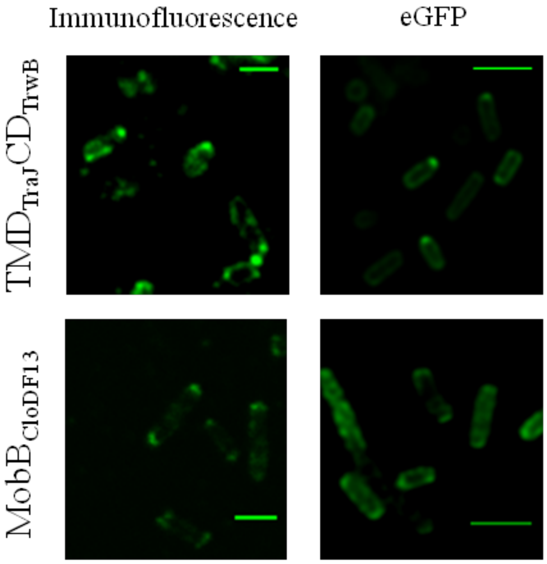

FIGURE S2. Subcellular location of $MobB_{CloDF13}$ and $TMD_{TraJ}CD_{TrwB}$ proteins visualized by immunofluorescence and eGFP fluorescence microscopy. The target proteins were expressed in *E. coli* BL21C41(DE3) strain by induction at OD_{600} 0.4 with 1 mM IPTG 20 hours at 25°C. For immunofluorescence detection cells were immunostained with mouse anti-His (C-term) monoclonal antibody as primary antibody, and Alexa Fluor goat anti-mouse as secondary antibody. Image acquisition was performed in an Olympus FluoviewTM 500 confocal fluorescence microscope.

Images of eGFP fluorescence microscopy were acquired in a Leica TCS SP5 confocal fluorescence microscope, with a 60X oil immersion objective. Sample excitation was performed with 488 nm wavelength, while fluorescence emission was measured between 505 and 525 nm. The images were analysed using Huygens and ImageJ softwares. Scale bar: 2 µm.
